# Case Report: The Alzheimer’s paradox: a clinically stable amnestic syndrome with full biomarker positivity and minimal imaging evidence

**DOI:** 10.3389/fmed.2025.1653232

**Published:** 2025-08-19

**Authors:** Anna-Chloé Balageas, Johnny Vercouillie, Nicolas Arlicot, Anne-Claire Dupont, Maria-Joao Santiago Ribeiro

**Affiliations:** ^1^Centre Mémoire Ressources et Recherche CHU Tours, Tours, France; ^2^UMR, iBrain, Université de Tours, Inserm, Tours, France; ^3^Radiopharmacy, CHU Tours, Tours, France; ^4^Nuclear Medicine, CHU Tours, Tours, France

**Keywords:** PET imaging, MCI, Alzheimer, neuroimaging, biomarker

## Abstract

We report the case of a 75-year-old patient with a clinically isolated and stable amnestic syndrome over fifteen years, despite a cerebrospinal fluid (CSF) profile strongly consistent with Alzheimer’s disease (AD). Serial PET imaging over two years showed only limited amyloid accumulation, no significant tau deposition, no hypometabolism, and no neuroinflammation. This case illustrates a striking dissociation between biomarker positivity and clinical progression, raising important questions about individual resilience and the prognostic value of AD biomarkers in isolation.

## Introduction

Alzheimer’s disease (AD) is typically diagnosed based on a combination of clinical symptoms and biomarker evidence (1). However, atypical presentations or prolonged stability despite pathological biomarker profiles challenge current diagnostic paradigms. We present a rare case of a clinically stable amnestic Mild Cognitive Impairment (MCI) with positive AD CSF biomarkers and minimal progression on multimodal PET imaging.

### Case description

The patient is a 75-year-old woman with a maternal history of Alzheimer’s disease. She has been followed by the Department of Psychiatric Neuropsychology for over fifteen years for a clinically isolated and highly stable amnesic syndrome. She has no significant vascular or psychiatric comorbidities and has maintained functional autonomy throughout the follow-up period. Her initial cognitive complaints emerged in her late 60s and prompted formal neuropsychological evaluation. At the age of 70, cognitive testing revealed an amnestic Mild Cognitive Impairment (MCI). General cognitive assessment included the Mini-Mental State Examination (MMSE 25/30), and a targeted evaluation of anterograde memory using the RL/RI-16, a French test comparable to the Free and Cued Selective Reminding Test (FCSRT). Her score on the RL/RI-16 was 36/48, consistent with isolated verbal episodic memory impairment. Despite these deficits, her autonomy in daily living activities was preserved. Structural MRI at that time revealed subtle atrophy of the right hippocampus. Cerebrospinal fluid (CSF) analysis showed hallmark abnormalities consistent with Alzheimer’s pathology, including elevated total tau (706 ng/L), phospho-tau (99 ng/L), reduced β-amyloid 1–42 (789 ng/L), and a low Aβ42/Aβ40 ratio (0.039).

**Figure 1:**
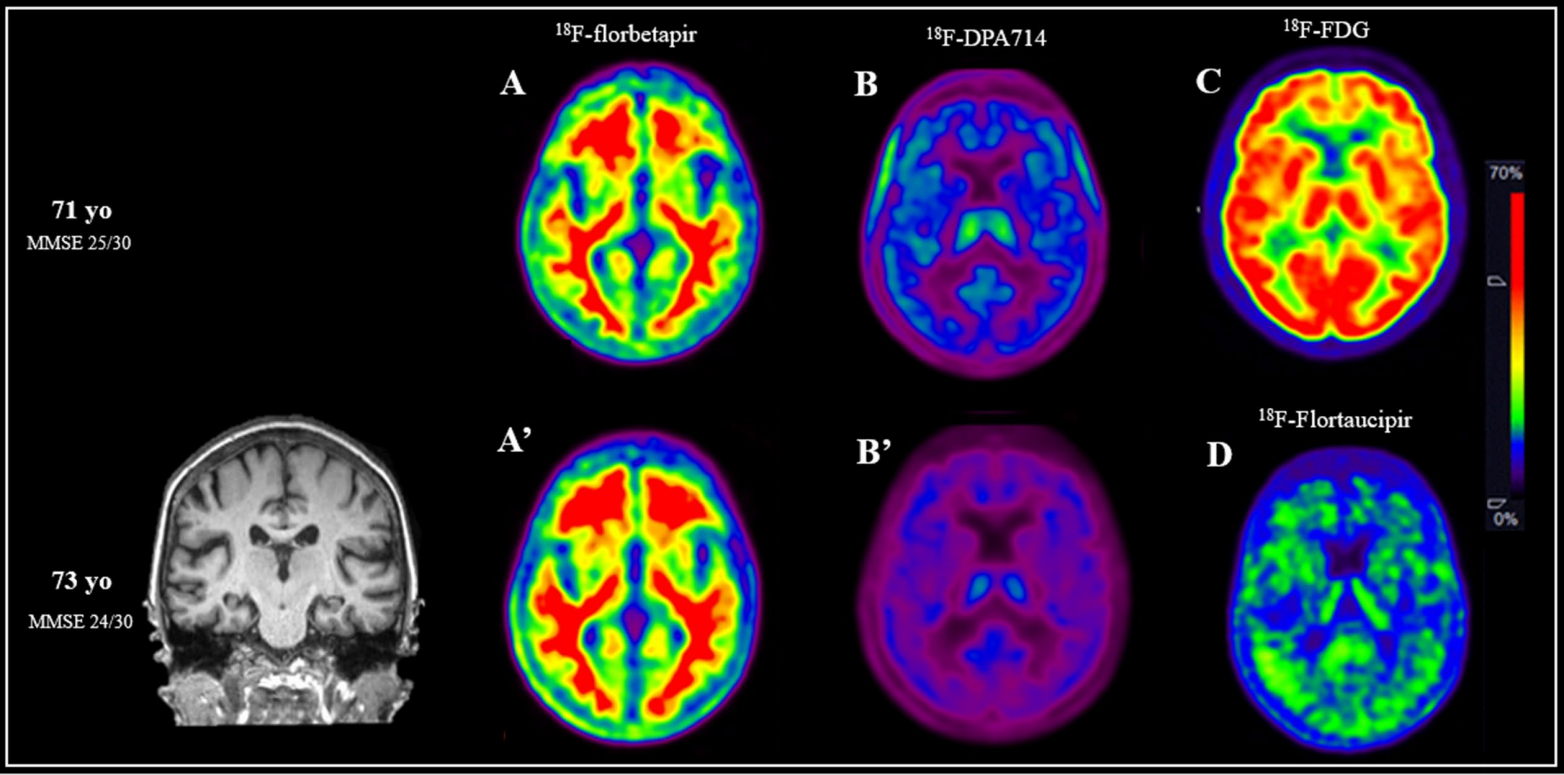
Multimodal PET imaging and cognitive assessments at ages 71 and 73 illustrate the progression of this patient with clinically stable amnestic mild cognitive impairment and biomarker-confirmed Alzheimer’s disease. At age 71, amyloid PET imaging using ^18^F-florbetapir **(A)** revealed moderate amyloid accumulation in the frontal cortex. Neuroinflammation imaging with ^18^F-DPA714 **(B)** showed no evidence of microglial activation, and ^18^F-FDG PET **(C)** demonstrated preserved cortical glucose metabolism, suggesting maintained neuronal activity. At that time, cognitive testing yielded a Mini-Mental State Examination (MMSE) score of 25/30. Two years later, at age 73, repeated amyloid PET **(A’)** showed a slight increase in frontal amyloid deposition. Neuroinflammation imaging **(B’)** remained negative for microglial activation, indicating continued absence of an active inflammatory process. Tau PET imaging with ^18^F-flortaucipir, performed for the first time at this stage, revealed subtle binding in the right temporal cortex **(D)**, consistent with early tau deposition. Cognitive evaluation at age 73 showed a slight decline in MMSE to 24/30.

Over a two-year period, the patient underwent several PET imaging studies using various fluorinated ligands to evaluate different aspects: cortical amyloid plaque accumulation (^18^F-florbetapir), neuronal activity (^18^F-FDG), tau protein density (^18^F-T807 or ^18^F-flortaucipir), and neuroinflammation (^18^F-DPA714) (2–5). At baseline, at age 71, scans revealed a moderate amyloid burden in the frontal cortex, without evidence of microglial activation or significant hypometabolism, suggesting preserved neuronal function and a lack of active neuroinflammation (Figures 1A–C). Cognitive assessment at that time showed a MMSE score of 25/30. Two years later, at age 73, the patient was re-evaluated using ^18^F-DPA-714, ^18^F-florbetapir, and, for the first time, ^18^F-flortaucipir. The amyloid load showed slight progression in the frontal cortex (Figure 1A’), consistent with the known amyloid trajectory, although amyloid distribution alone does not distinguish between typical and atypical forms of dementia. Microglial activity remained undetectable (Figure 1B’) reinforcing the absence of an active inflammatory process. Importantly, the new tau PET imaging revealed subtle binding in the right temporal cortex (Figure 1D), indicating early-stage tau deposition. At that time, the patient’s MMSE score had declined slightly to 24/30, reflecting a modest progression in global cognitive function.

## Discussion

This case presents an unusual clinical-biological dissociation: CSF biomarkers strongly suggest AD pathology, yet the patient exhibits minimal imaging abnormalities and remains clinically stable over an extended period. The pattern of tau deposition observed—restricted to the right temporal cortex—is consistent with the expected medial-to-lateral temporal spread seen in early tau pathology, before involvement of broader neocortical areas. The absence of tau signal in regions such as the frontal cortex, where amyloid load had progressed, may reflect the slow pace of tau accumulation in this individual. Moreover, recent studies have demonstrated that changes in CSF Aβ42 levels often precede detectable amyloid plaques on PET imaging, and that CSF biomarkers may reflect more dynamic or soluble forms of pathology (6). This dissociation between biomarker progression and clinical symptoms supports the concept of brain resilience, wherein underlying pathological changes do not immediately result in functional decline. This study has several limitations. The absence of a structural MRI scan at the time of baseline PET imaging limits anatomical correlation, particularly with respect to emerging tau signal. Additionally, tau PET was not available at baseline, preventing assessment of its progression.

Diagnosing AD can be difficult, as atypical presentations—such as language, visual, and frontal variants—can occur alongside the classic amnestic form (7). These findings underscore the complexity of early AD diagnosis and caution against over-reliance on biomarker profiles without longitudinal clinical context.

## Data Availability

The original contributions presented in the study are included in the article/supplementary material, further inquiries can be directed to the corresponding author.
